# The write way to spell: printing vs. typing effects on orthographic learning

**DOI:** 10.3389/fpsyg.2014.00117

**Published:** 2014-02-13

**Authors:** Gene Ouellette, Talisa Tims

**Affiliations:** ^1^Department of Psychology, Mount Allison UniversitySackville, NB, Canada; ^2^Department of Psychology, Dalhousie UniversityHalifax, NS, Canada

**Keywords:** orthographic learning, lexical representations, self-teaching, spelling, reading, literacy, printing, typing

## Abstract

Prior research has shown superior orthographic learning resulting from spelling practice relative to repeated reading. One mechanism proposed to underlie this advantage of spelling in establishing detailed orthographic representations in memory is the motoric component of the manual movements evoked in printing or writing. This study investigated this contention directly by testing the effects of typing vs. printing on the orthographic learning achieved through spelling practice, and further evaluated whether practice modality interacts with pre-existing individual characteristics. Forty students in grade 2 (mean age 7 years 5 months) were introduced to 10 novel non-words. Some of the students practiced spelling the items by printing, while the others practiced spelling them on a keyboard. Participants were tested for recognition and spelling of these items 1 and 7 days later. Results revealed high rates of orthographic learning with no main effects of practice modality, testing time, or post-test modality. Hierarchical regression analyses revealed an interaction between typing proficiency and practice modality, such that pre-existing keyboarding skills constrained or facilitated learning within the typing-practice group. A similar interaction was not found between printing skills and learning within the printing group. Results are discussed with reference to both prominent reading theory and educational applications.

## Introduction

When children enter into the often arduous task of mastering early literacy skills, they begin applying their knowledge of the alphabet by mapping speech sounds onto letters. Such letter-sound associations underlie early literacy as children sound out words in learning to read, and conversely analyze the sounds in words to create a spelling attempt. But as children progress on the pathway to literacy, they become better able to recognize words fluently with far less apparent effort and to spell words correctly by conventional standards. There thus appears to be a transition in literacy acquisition from a reliance on more laborious phonologically based sounding out strategies to the use of memory representations for longer letter patterns and entire words (Ehri, 2005). These memory representations are referred to as orthographic representations and the process of storing such representations as orthographic learning. There is now considerable evidence that orthographic representations are stored as a result of print exposure during decoding practice, resulting in a growing corpus of representations to be used in subsequent reading and writing activity (e.g., Share, 2004; Castles and Nation, 2006). More recently, spelling practice has been found to result in superior orthographic learning, relative to print exposure through reading alone (Conrad, 2008; Ouellette, 2010), although the reason for this has not been established. The present study evaluates the role of one component of the spelling process often hypothesized to underlie this advantage, i.e., the manual movements involved in printing, while also evaluating the effectiveness of computer keyboarding for learning new orthographic representations. In the process, we further consider whether any modality effects interact with individual characteristics to support or constrain orthographic learning.

This research draws on two presently distinct bodies of literature: one dealing with orthographic learning and the other with effects of printing vs. keyboarding in establishing lexical representations in long term memory. Indeed one goal of this research is to bring these two areas of study together, in evaluating the role of the modality used in spelling practice and how this may interact with individual learner characteristics, when it comes to learning new word representations. To the best of our knowledge this has yet to be specifically tested within an experimental orthographic learning paradigm, yet is especially important when one considers the prominence prescribed to orthographic learning in developmental literacy theory, the possible involvement of motor commands/patterns in establishing lexical representations, the increasing use of computers within the home and classroom, and the diversity seen across learners.

## Orthographic learning and spelling

As just outlined, orthographic learning allows a student to progress from less efficient sounding-out strategies to the use of word-specific memory representations. Much developmental theory explains fluent reading and accurate spelling as hinging upon such representations. Ehri (2005) has provided a now well cited descriptive theory for instance, that depicts the beginning reader/speller as one who progresses through phases of proficiency related to their developing alphabetic and phonological knowledge. Through experience with print, longer and longer letter strings become stored in memory. Children in the final “consolidated alphabetic phase” are able to read fluently and to spell accurately, by relying upon these stored orthographic representations.

According to Ehri (2005), orthographic learning comes about through experience with printed language. The importance of sounding out words or phonological recoding in orthographic learning is further detailed in Share's (1995, 2004) self-teaching hypothesis. Share proposes that it is the process of applying letter-sound knowledge in decoding printed text that allows the reader to store longer and more detailed representations for encountered words. It is through decoding that children in essence, teach themselves word-specific representations, which are then available for future encounters with these now learned words. As in Perfetti and Hart's (2002) lexical quality hypothesis, proficient reading and spelling are seen to rely upon such highly refined lexical representations. While Share, along with Perfetti and Hart, posits an item-based theory rather than one of developmental phases, the focus on orthographic learning is the same. There have been a number of recent studies in support of Share's hypothesis, showing that elementary school aged children learn word specific orthographic representations from reading both contextual passages and isolated words (e.g., Nation et al., 2007; Ouellette and Fraser, 2009; Wang et al., 2011). Much of this research has employed an orthographic learning paradigm modeled after Share's (1995, 1999) earlier work. The basic paradigm involves exposing children to ambiguously spelled non-words. The use of non-words controls for effects of previous exposure, and the spellings are ambiguous in the sense that the pronunciation could be matched to more than one possible spelling (e.g., *yait* which could conceivably also be spelled as *yate*). Following a series of practice trials in which these non-words are read, participants are tested for spelling and/or recall with a forced choice task where one of the choices is a homophone foil (i.e., the alternate yet plausible spelling). If a participant persists with a phonologically based approach, they would be as likely to spell or identify the homophone as they would the practiced item. Success on these post-tests is thus seen as reflecting orthographic learning, as accurate identification or spelling reflects a newly stored memory representation.

Following the lead of Shahar-Yames and Share (2008), Ouellette (2010) modified the orthographic learning paradigm just described to replace the repeated readings for some students with spelling practice. English-speaking students in grade 2 were randomly assigned to either a traditional orthographic learning condition involving reading or to one where the reading was replaced with repeated spelling to dictation. The auditory and visual exposures to the non-words were carefully controlled across conditions, yet Ouellette found that the children in the spelling practice group outperformed the other students on post-tests administered 1 and 7 days after the practice session. Similar results have been reported for Hebrew speaking grade 3 students (Shahar-Yames and Share). It thus appears that spelling practice provides a superior milieu for orthographic learning relative to print exposure garnered through reading alone. This contention is further supported by Conrad (2008) who directly compared reading and spelling practice and the transfer between the two skills with grade 2 students. Employing a list of real words with shared orthographic rime units, Conrad reported that representations learned through one skill transferred to the other, as the students were better able to spell words they had practiced reading, and to read words they had practiced spelling. Importantly, the transfer was greater from spelling practice to reading (than from reading practice to spelling). There was also transfer within each skill to untrained words, but this was again greater for spelling than for reading.

The research just reviewed points to superior orthographic learning through spelling compared to reading practice, further establishing the relevance and importance of spelling practice in establishing lexical representations for use in subsequent literacy tasks. What remains uncertain is the mechanism behind this effectiveness of spelling practice. In comparing the exercise of spelling to that of reading, one salient difference is the motoric component of the manual movement involved in writing out words. There has long been a notion that there is something special about the manual movements involved in printing or writing by hand that aids in memory encoding and/or retrieval, suggesting a possible motoric component to lexical representations (Masterson and Apel, 2006). Indeed, multi-sensory teaching approaches are very much based on this premise (Hulme, 1983; Hulme and Bradley, 1984).

## Printing by hand vs. keyboarding in spelling and literacy learning

The possible role of a motoric component in establishing representations for literacy has been directly tested in the past in a small number of studies that have compared the effects of printing vs. keyboarding on specific learning outcomes. Research with children just learning the alphabet for example, has shown superior letter learning following printing practice relative to keyboarding (Longcamp et al., 2005, 2008). This has led Longcamp and colleagues to propose that memory representations of letters incorporate visual and motor information across a complex neural network (Longcamp et al., 2005, 2008). Indeed, similar brain regions, specifically Broca's and areas of bilateral inferior parietal lobes, have been implicated in both printing by hand and visual letter recognition (Longcamp et al., 2005). It remains uncertain whether a similar role of motoric knowledge exists for longer, more refined lexical representations as there have been few studies that have focused on modality effects for learning more complex orthographic representations; we next turn our attention to this limited extant literature.

The seminal study directly comparing effects of printing and keyboarding on learning to spell and read was reported by Cunningham and Stanovich (1990), as motivated by the earlier work of Hulme and Bradley (1984). Cunningham and Stanovich gave grade 1 students practice spelling 30 words over 4 consecutive days. The words were randomly split into two lists of 15 words, and each participant practiced each list on 2 days of the week (1 list Monday and Wednesday, the other Tuesday and Thursday). Post-testing on reading and spelling was completed on the Friday. Spelling modality was manipulated within subjects, as five words on each list were spelled by printing, 5 by typing on a keyboard, and 5 by arranging letter tiles. Post-tests revealed superior spelling accuracy for words practiced through printing, although there was no effect of practice modality on reading accuracy. It should be noted that the words used in this study varied in terms of sound-letter consistency; most could be read by sounding out and blending and at least some of the words could be spelled accurately by sounding out rather than relying on orthographic representations (e.g., man, help). The participants were also only in grade 1, at an age when phonological strategies may be more appropriate and spelling skills unstable (Masterson and Apel, 2006). Further, the use of real words raises concerns about previous exposure and pre-existing orthographic knowledge. Thus it is not clear as to whether these oft cited results can be confidently interpreted with respect to modality effects on orthographic learning.

To address the issue of previous exposure and pre-existing orthographic representations, Vaughn et al. (1992) replicated Cunningham and Stanovich's (1990) study with first graders, but this time the children were pre-tested on the word lists and any known words were discarded. In all, 21 of the original 30 stimulus words had to be replaced as they were spelled correctly at pre-test by at least one student. In contrast to the findings from the original study, Vaughn et al. found no significant differences in learning across the spelling modality conditions, leading them to conclude that printing by hand was not a superior milieu for learning to read and spell. Adding further support to this conclusion, Vaughn and colleagues completed another replication, adding individualized feedback to increase learning, and reported the same null results (Vaughn et al., 1993).

Although the research conducted by Vaughn et al. (1992, 1993) controlled for pre-existing word knowledge, the methodology still suffered from the same limitations raised earlier for Cunningham and Stanovich's (1990) original study. In particular, the variability in word consistency makes the results difficult to interpret with respect to the important developmental skill of orthographic learning. Further, all of these studies have taught quite a large corpus of words, allowing only two practice trials per word, to young grade 1 students. As result, learning rates were quite low across studies. In addition, it is important to note that in the methodology detailed by Vaughn et al., students copied the spellings rather than deriving them from memory. This is important as the benefits of spelling practice have been proposed to be related to the process of analyzing a word and retrieving information from memory in generating the spelling (Ouellette and Sénéchal, 2008; Sénéchal et al., 2012); copying may not provide for the same deep level of processing and this could have also contributed to the low learning rates and null results reported in this research. When these concerns are taken together, the present literature cannot establish whether there are modality effects on orthographic learning beyond single letter learning, and hence the mechanisms that underlie the effectiveness of spelling practice in learning word forms remain elusive.

## The present study and the role of individual differences

In considering the limited research comparing the effects of printing to keyboarding in learning longer orthographic representations for spelling and reading, the results are clearly equivocal. Together this literature paints an unclear picture and most importantly, methodological concerns prevent the interpretation of results with respect to the role of spelling modality in orthographic learning, an issue of significant theoretical and practical significance. The present study aims to address this issue by directly comparing the effects of spelling practice through printing and keyboarding, within a carefully designed orthographic learning paradigm as described earlier and adapted to include spelling practice (as per Shahar-Yames and Share, 2008; Ouellette, 2010). The use of ambiguously spelled non-words allows for the specific evaluation of orthographic learning while also controlling for previous exposure, and the present study involves a sample of students in Grade 2, a grade level where orthographic learning would be especially relevant in making the transition to more fluent reading and accurate spelling (Conrad, 2008; Ouellette, 2010).

The present study also incorporates a number of other important methodological improvements over the extant literature. Primarily, the word set is restricted to 10 items, and each is practiced four times, allowing more opportunity for orthographic learning to occur than what has been reported in the past. Further, the spelling practice implemented requires the participant to spell to dictation after being exposed to the correct spellings rather than just copying the items, thus providing for a more analytic process and potentially deeper level of processing. We also incorporate a counterbalanced design with respect to the modality used in post-testing. Although research with older students has shown performance on spelling assessment not to be affected by the modality used (printing or keyboarding) in the administration of the test (Masterson and Apel, 2006), it is important in research that post-test methodology not resemble one training condition more so than another. Therefore, half the non-words practiced are assessed at post-test in the same modality as practiced, while the others are assessed in the opposite modality.

The present research also includes a pre-test battery to assess baseline levels of printing, typing, reading and spelling proficiency, allowing for the evaluation of the effects of pre-existing skills in these areas on orthographic learning. Of particular interest in the present study are possible interactions between individual differences in terms of pre-existing skills and the modality used to practice the new spellings. In other words, do particular students benefit more from practice in one modality over the other (or conversely, are some hindered within one modality more than the other)? One area that may be hypothesized to interact with the practice modality used for spelling is printing and typing ability. In the research reviewed earlier that compared printing with typing, children's baseline skills for printing and typing were not assessed. Yet it may be reasonable to hypothesize that success with learning through practice in either modality may depend at least in part upon the skills that children bring with them into a study, especially considering that printing and typing skills appear to develop independently and there is considerable variability in these skills across children (Berninger et al., 2006).

While typing proficiency has yet to be examined with respect to its impact on early spelling practice, some have proposed slow or laborious printing to limit written composition and spelling by tapping cognitive resources and straining working memory; indeed, printing fluency has been shown to be correlated with spelling in the early grades (Kim et al., 2014), although whether this impacts orthographic learning specifically is not certain. In a study evaluating modality effects on spelling for students with spelling disabilities, Berninger et al. (1998), in accord with the studies of Vaughn et al. (1992, 1993), reported an overall null result in comparing effects of printing and keyboarding in spelling instruction. Interestingly, these researchers also assessed printing skills to evaluate a possible interaction between printing proficiency and practice modality, yet did not find any such interaction within their data. Berninger et al. did not assess keyboarding skills however, and it is not certain if their results apply to a general population of early learners. Further, many of the concerns surrounding word consistency and familiarity raised previously apply to the Berninger et al. research as well. Accordingly, it remains uncertain if pre-existing printing and typing skills do indeed interact with practice modality when it comes to orthographic learning.

The present study has been designed to address a prominent gap in current research. The information garnered here stands to add to current theory and to inform teaching practice. The topic of study is of special relevance given the advancement of computer technology and applications into the home and classroom (see Blok et al., 2002) and the prominence of the self-teaching hypothesis (Share, 2004).

## Methods

### Participants

Forty-four Grade 2 students from an elementary school in a small Canadian town participated. Three children were absent on the day of the first post-test and were therefore excluded from the final sample. One student was identified as both a univariate (*z*-scores >3.0) and multivariate (through scatterplots) outlier on a number of pre-test measures and was also excluded from the final sample. Thus, a total of 40 children (18 males and 22 females) with a mean age of 7.42 years (*SD* = 0.26) were included in the analysis reported here. Of these children, 27.5% had a parent with a post-graduate degree, 32.5% had a parent with an undergraduate university degree, 17.5% had a parent who had attended college, 20% had a parent whose highest level of education was high school, and 2.5% had a parent who had not completed high school. All participants were English speaking with no history of speech, language, or learning difficulties.

### Materials: initial assessment

#### Word reading and decoding

The Test of Word Reading Efficiency (TOWRE; Torgesen et al., 1999) was administered as a measure of reading skills. This is a timed reading test in which participants have 45 s to read a list of words and receive a score based on how many words are read correctly. The test is repeated using a list of non-words. Many forms of reliability are reported, all of which are at or above 0.90.

#### Spelling

The Woodcock Johnson III (WJ-III; Woodcock et al., 2001) spelling subtest was administered. Children were asked to spell letters and words that increased in difficulty. Testing continued until six consecutive errors were made or until the participant reached item 59. Many forms of reliability are reported for this test, with a median of 0.90.

#### Baseline printing and typing skills

To obtain baselines of printing and typing proficiency, non-standardized tests based on previous research were administered (Berninger et al., 2006; Masterson and Apel, 2006; Kim et al., 2014). Children were asked to copy the passage “Are you amazed at how much you have learned so far? Just how high to build your speed is the next question,” by typing and by printing. This passage is often used to assess typing as it contains nearly all letters on the keyboard. Children were also asked to produce both capital and lowercase letters of the alphabet in order by typing and by printing (e.g., Aa, Bb, Cc). In all tasks children were given 60 s to complete the test. These tasks were scored by counting the number of correctly produced characters to achieve a characters-per-minute score to reflect automaticity and proficiency in these areas. For the printing tasks, the letters had to be identifiable out of context and only reversals that could not be confused with other letters were accepted as correct. Inter-rater reliability was excellent (0.97).

### Materials: training stimuli

Participants were trained on 10 non-words used in previous research (see Bowey and Miller, 2007; Ouellette, 2010). These 10 non-words are ambiguous such that there is more than one possible spelling using a phonetic approach (see the Appendix).

### Procedure

Children were first administered the pre-tests to obtain information about their skills prior to the study. This was done in a quiet, empty room by a trained research assistant. Children were administered the tests in one individual session, in the following order: typing passage, typing alphabet, TOWRE: Words, TOWRE: Non-words, WJ-III: Spelling, printing passage, printing alphabet. Half of the participants had the reverse order of the typing and printing tasks (i.e., the two printing tests at the start of the session and the typing tasks at the end).

Following completion of all individual assessments, each child received a training session in which they practiced spelling the 10 non-words in their assigned practice modality (printing or typing). Modalities were randomly assigned within each classroom, such that half the children from each class were assigned to each of the two conditions. The 10 non-words were typed on index cards and presented one at a time at the start of the practice session for the child to read aloud (visual exposure). Each card was in view for 5 s and any errors were corrected with a model to repeat. Once all the words had been read, the practice trials began. The index cards were shuffled and the child was once again shown a card and asked to read the non-word aloud. The card was removed from view after 5 s; following a 5 s pause, the child was asked to spell the same non-word in a dictation (i.e., following a pronunciation by the researcher). Children in the printing condition spelled the non-word with a pencil on a blank index card; those in the typing condition used a standard PC keyboard. Typed spellings were displayed on the monitor within Microsoft Word, with the font set at 24 point (Arial) to make the character size approximately equivalent to those produced in the printing group. If the item was spelled correctly, the child was asked to read it aloud once more and then the spelling was immediately removed from view (the card flipped over for the printers and the computer screen cleared for the typers). If the child's spelling was incorrect, the original card was shown for the child to read. Regardless, the child was then asked to spell the word a second time. Once more, they read their spelling (if correct) or the original stimulus card (if incorrect), and the spellings were removed from view immediately. In all, children saw, read, heard, spelled, read, heard, spelled, and read each item on each trial. This procedure was followed until the entire deck of index cards was completed twice. It may be most accurate to describe this practice as spelling *plus* reading rather than as just spelling. Separating spelling from reading would jeopardize ecological validity (Conrad, 2008; Shahar-Yames and Share, 2008), and thus the spelling practice here deliberately incorporated reading as would be the natural occurrence.

All children were individually tested both one and 7 days later with a multiple-choice identification test and a spelling to dictation test. The multiple-choice test involved 10 items, one for each non-word, which included four different choices. The choices included the target and a (pseudo)homophone, as well as two other choices that were visually similar and/or contained the same letters but in a different order. The child was instructed to circle the correct spelling of the target word for each of the ten items. In the spelling test, the researcher simply dictated the target non-words for the child to spell. No feedback was provided in either task.

The design was fully crossed with respect to post-test spelling modality. This means that all participants were tested for spelling on half the words in their trained modality and on half the words in the other modality. The words were thus split into two lists, with vowel patterns matched across lists (see Appendix). Additionally, post-test modality was counter-balanced across lists, such that children in each practice group printed List A and typed List B, while others typed List A and printed List B. The design was fully counterbalanced.

#### Results

A Principle Components Analysis was run with Direct Oblim Rotation on the multiple measures of Printing (Alphabet and Passage) and Typing (Alphabet and Passage) as well as the two TOWRE subtests (words and non-words) to explore possible data reduction by combining these into three composite scores (printing, typing, reading). However, the passage measures were unable to load on one factor and instead split between the three. The analysis was rerun without the Printing and Typing Passage tests (as the semantic and syntactic complexity of the phrase used was thought to have influenced performance) and this resulted in three factors with simple structure accounting for 97% of the variance. All loadings were >0.95. Therefore, only the Alphabet tests were used in the following analyses as indices of printing and typing skill, and the two TOWRE tests were combined to create a reading composite.

Descriptive statistics for the initial assessment of printing, typing, and literacy skills are provided in Table 1 along with decoding accuracy for the first exposure to the training stimuli (from the start of the training session). A multivariate analysis of variance indicated no significant differences between the two groups on any measure (all *F*s < 1.01; *p*s ranged from 0.32 to 0.90). Thus, the children in each group had comparable skills prior to the practice session.

**Table 1:** **Initial assessment performance as a function of practice group**.

		**Practice group**	
		**Printing (*n* = 19)**	**Typing (*n* = 21)**
	**Max.**	**Mean (*SD*)**	**Mean (*SD*)**
Reading composite	167	79.74 (26.39)	78.71 (24.93)
Spelling	59	25.26 (4.74)	24.24 (3.65)
Printing baseline	52	24.74 (9.72)	23.38 (8.57)
Typing baseline	52	16.89 (6.38)	18.43 (7.26)
Stimuli decoding	10	7.95 (2.12)	7.24 (2.85)

Note. Max., maximum score possible.

The first objective of this research was to investigate the effect of practice modality (printing vs. typing) on orthographic learning. Table 2 presents the proportions of practiced words identified correctly during the recognition post-tests, as a function of practice modality and post-test time (1 and 7 days). Accuracy rates were high but not at ceiling levels, hovering around 80% across groups and test dates. To investigate whether performance on the recognition tasks differed between the spelling practice groups or testing dates, a 2 (Training group: printing vs. typing) × 2 (Time: day 1 vs. day 7 post-test) repeated-measures analysis of variance (ANOVA) was conducted. There was no significant effect for group, *F*_(1, 38)_ ≤ 1.00, *p* = 0.86 or for time, *F*_(1, 38)_ = 1.29, *p* = 0.26. The interaction between time and training group was also not found to be significant, *F*_(1, 38)_ ≤ 1.00, *p* = 0.62.

**Table 2:** **Proportions of target non-words selected on recognition post-tests**.

	**Practice group**	
	**Printing (*n* = 19)**	**Typing (*n* = 21)**
	**Mean (*SD*)**	**Mean (*SD*)**
Recognition Day 1	0.82 (0.17)	0.80 (0.18)
Recognition Day 7	0.78 (0.18)	0.78 (0.19)

The second and more stringent post-test of orthographic learning required participants to spell the practiced non-words in a dictation. Table 3 presents the means and standard deviations of the proportion of non-words spelled correctly by each practice group, across post-test days and post-test modality. Recall that within each group half of the items were post-tested via printing, the other half through typing. Again, accuracy rates appear consistent across groups and time, as well as across post-test modalities. A 2 (Training group: printing vs. typing) × 2 (Time: day 1 vs. day 7 post-test) × 2 (Post-test modality: printing vs. typing) repeated-measures ANOVA was conducted to evaluate this pattern of results. As suggested by the data presented, there was no significant main effect for group, *F*_(1, 38)_ ≤ 1.00, *p* = 0.99, time, *F*_(1, 38)_ ≤ 1.00, *p* = 0.71, or for modality used in the post-test, *F*_(1, 38)_ ≤ 1.00, *p* = 0.46. There were also no significant interaction effects. Thus, groups responded similarly across post-test modality and days, with both groups showing impressive orthographic learning.

**Table 3:** **Proportions of target non-words spelled correctly on post-tests**.

	**Practice Group**	
	**Printing (*n* = 19)**	**Typing (*n* = 21)**
	**Mean (*SD*)**	**Mean (*SD*)**
**SPELLING DAY 1**		
Assessed via printing	0.63 (0.27)	0.65 (0.28)
Assessed via typing	0.69 (0.26)	0.62 (0.29)
**SPELLING DAY 7**		
Assessed via printing	0.60 (0.25)	0.69 (0.24)
Assessed via typing	0.69 (0.29)	0.66 (0.29)

The next research question concerns the possible role of individual differences in literacy, printing, and typing skills on orthographic learning. In particular, it is of interest to explore whether skills in any of these areas interacted with the practice modality, which would suggest one modality may be more preferable over the other for certain students. This was addressed with multiple regression analyses in which individual data from the pre-tested areas served as predictor variables and performance on the post-tested recognition and spelling tests served as criterion variables. Given the null results reported above, data was collapsed across test dates and also across post-test modalities for the spelling tests. Preliminary analysis revealed that only pre-tested reading and spelling levels directly predicted the overall orthographic learning outcomes, and hence these literacy skills were entered in the first step of the models. Practice group was dummy- coded, and interaction terms were created by multiplying the dummy coded variable with each of the assessed areas. These interaction terms were tested individually in the last step of the regression models. All models are presented in Table 4.

**Table 4:** **Regression analysis predicting performance on multiple-choice and spelling post-tests**.

	***R*^2^**	**Δ*R*^2^**	**Δ*F***
**CRITERION: RECOGNITION TASK**			
1. Literacy (Reading and spelling)	0.516	0.516	19.66^***^
2. Practice group	0.516	0.000	<1.00
3. Reading × group interaction	0.517	0.001	<1.00
3. Spelling × group interaction	0.516	0.000	<1.00
3. Printing × group interaction	0.517	0.001	<1.00
3. Typing × group interaction	0.571	0.055	4.51^*^
**CRITERION: SPELLING TASK**			
1. Literacy (Reading and Spelling)	0.565	0.565	24.01^***^
2. Practice group	0.568	0.003	<1.00
3. Reading × group interaction	0.574	0.006	<1.00
3. Spelling × group interaction	0.576	0.008	<1.00
3. Printing × group interaction	0.569	0.001	<1.00
3. Typing × group interaction	0.648	0.080	7.96^**^

* p < 0.05,

** p < 0.01,

*** p < 0.001.

In the first models presented in Table 4, the criterion variable was performance on the recognition tasks. Entered in step 1, children's pre-existing literacy skills accounted for a sizeable 51.6% of the variance in post-test recognition scores. Adding the practice group coding in step 2 did not account for any additional variance, consistent with the ANOVA results. Adding interaction terms separately at step 3 did not add any explanatory power to the model, except in the case of the term involving typing skills: the addition of a term modeling the interaction between typing ability and group assignment accounted for an additional 5.5% of the variance in orthographic recognition, bringing the total variance accounted for to an impressive 57.1%.

**Figure 1:**
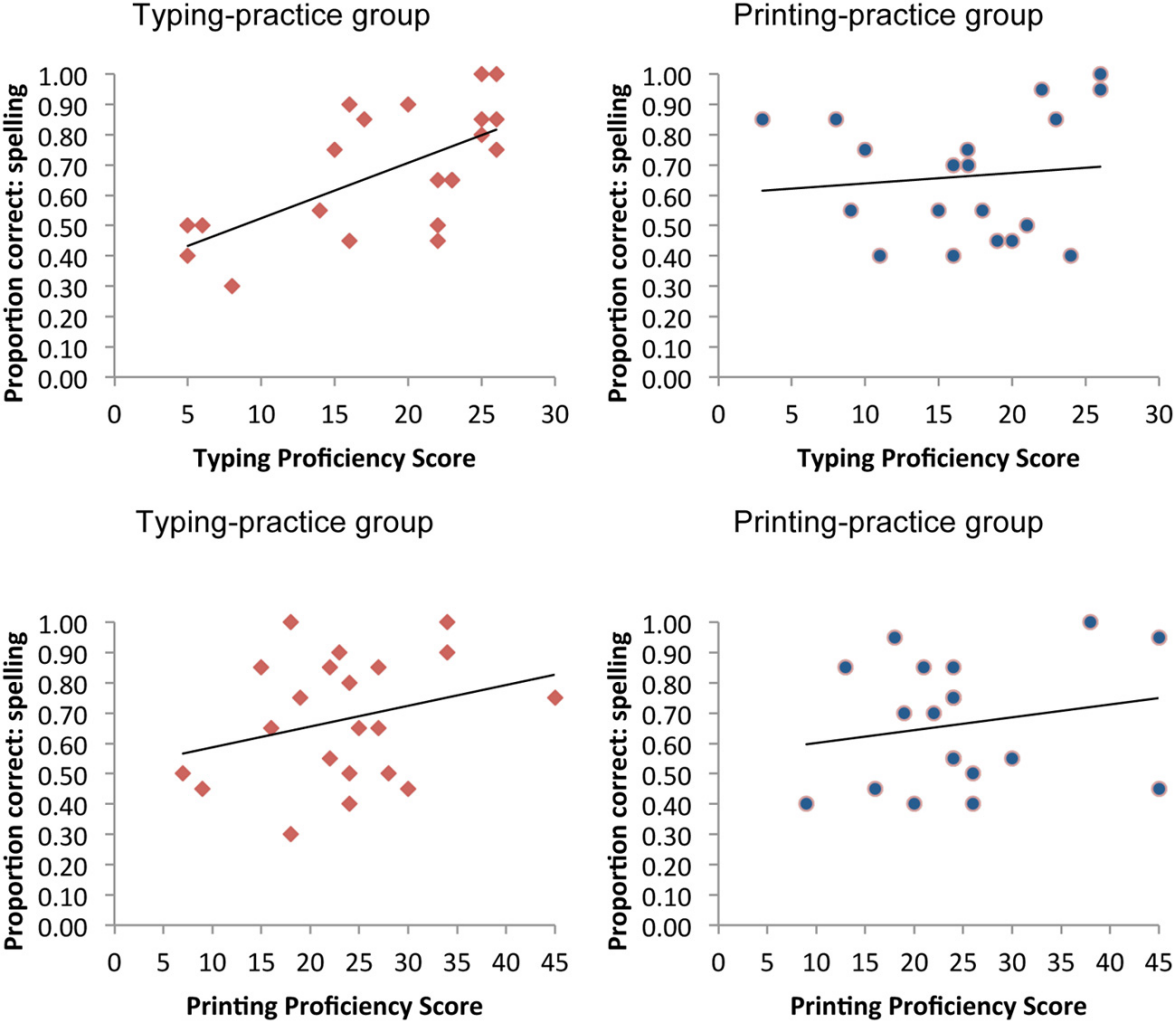
**Scatterplots of the relations between pre-tested areas and performance on the spelling post-tests, as a function of practice group.** The upper panels depict the nature of the interaction between typing skills and practice group; the lower panels show the absence of any interaction between printing skills and practice group.

The bottom half of Table 4 shows the regression results with total post-tested spelling performance as the criterion variable. Pre-existing literacy skills accounted for a significant 56.5% of variance in spelling post-test performance. Entering practice group assignment in step 2 did not account for any additional variance, once again consistent with the ANOVA results. The only interaction term to make a significant contribution to the model was again found to be one incorporating pre-tested typing proficiency: the typing interaction term accounted for an additional 8.0% of unique variance in spelling post-tests, bringing the total variance explained to 64.8%. The pattern of results behind this significant interaction term is depicted clearly in Figure 1. From these scatterplots, it is apparent that typing skills facilitated and/or constrained learning but only within the typing practice group. For comparison purposes, the lower panels in the Figure show how a similar influence of printing skills was not observed within the printing practice group^1^.

## Discussion

The present study evaluated the influence of manual printing on establishing orthographic representations in memory, by comparing practice modality effects on the orthographic learning that occurs through spelling practice. To the best of our knowledge we are the first to address this research question by employing a carefully devised orthographic learning paradigm in which grade 2 students practiced spelling novel non-words either by printing or by typing. The non-words, as used in previous research, had ambiguous spellings and thus success in learning these new forms is seen as a clean metric of orthographic learning. This methodology then, makes it possible to specifically isolate the effects of printing practice vs. typing practice on the learning of new representations. The results indicated that spelling practice via printing and typing led to comparable amounts of orthographic learning, as measured by both visual recognition and spelling post-tests. The only pre-existing participant characteristic that interacted with practice modality in influencing orthographic learning was found to be typing skills; printing skills did not interact with practice modality in a similar fashion.

To measure orthographic learning, the present research examined performance on a recognition task and a spelling task. While not at ceiling levels, performance was strong across these tasks, both 1 and 7 days following the practice session and greater than what has typically been reported in the past (Cunningham and Stanovich, 1990; Vaughn et al., 1992, 1993). Thus, it appears that the methodology employed here was successful in bringing about orthographic learning, adding validity to the reported findings. The present research is the first to compare the effects of printing and typing utilizing an orthographic learning paradigm with a constrained set of ambiguously spelled non-words; previous studies have used a larger corpus of real words varying in consistency as well as younger participants and a procedure that included copying rather than devising spellings from memory. All of these factors may well have contributed to insufficient learning and the conflicting results of past research.

While the current results (of successful orthographic learning) support the use of spelling as a self-teaching mechanism (Share, 1995, 2004; Ouellette, 2010), they do not support the hypothesis that spelling's effectiveness is linked to the manual movements involved in printing (Hulme, 1983). Given the methodological care of the present study, there is reason to have confidence that the lack of between-group differences in orthographic learning reported here is a valid and important finding in itself and makes an important contribution to both theory and teaching practice. That is, the null findings for any between-group differences suggest that printing and typing bring about equivalent levels of orthographic learning at this phase of literacy acquisition, confirming the earlier (null) findings of Vaughn and colleagues (1992, 1993) and Berninger et al. (1998) but with a more rigorous experimental design that specifically targeted orthographic learning. This may ease concerns of using keyboards within literacy curricula, while also clarifying the role of motoric knowledge and manual printing motions in learning; while there is evidence to suggest these may be important in initial alphabet learning where visual shape and motoric information appear connected (Longcamp et al., 2005, 2008), the current findings add to the literature showing no such connection for larger more detailed orthographic representations. The present results, in accord with Masterson and Apel (2006), suggest that lexical representations utilized in spelling are modality-free in terms of stored detail.

## Individual differences in orthographic learning

The present research design importantly allowed for an evaluation of the effects of pre-existing individual differences on orthographic learning, as we obtained measures of literacy, printing, and typing proficiency at the onset of the study. Regression analyses indicated a primary role of pre-existing reading and spelling skills in orthographic learning. This is consistent with other research that has found skills such as decoding and orthographic knowledge to be significant predictors of orthographic learning (Castles and Nation, 2006; Ouellette and Fraser, 2009). Pre-existing literacy skills very much facilitate and/or constrain the acquisition of new orthographic representations regardless of the practice modality employed, highlighting the stability of early individual differences in literacy and the importance of early identification and intervention efforts.

An important novel finding of the present study was the significant interaction between typing skills and practice modality, such that pre-existing typing skills constrained and/or facilitated success in learning new words through typing practice; the same effect was not found for pre-existing printing skills on learning new words through printing. In other words, within the typing group only, orthographic learning was facilitated or constrained by pre-existing typing skills, even after controlling for pre-existing reading and spelling levels. While keyboarding skills have previously been found to interact with overall writing quality for older students in terms of content and style (e.g., Russell, 1999), this is the first study to show such an interaction with learning new orthographic representations. What makes this finding all the more interesting is that a comparable relation was not found between pre-existing printing skills and performance within the printing-practice group, a finding similar to that reported for spelling disabled students by Berninger et al. (1998), but somewhat surprising given past correlations between printing fluency and spelling (Kim et al., 2014). In the present study, weaker printing skills did not appear to constrain learning within the printing group and stronger printing skills did not facilitate learning. Thus the contention that weaker hand writers may benefit more from keyboarding (Russell, 1999; Blok et al., 2002) may not be empirically supported when it comes to learning new orthographic representations.

These results raise a pertinent question: why would orthographic learning be influenced by typing proficiency but not by printing skill? Printing fluency has been proposed to potentially interact with spelling in so far as laborious printing would tap cognitive resources and strain working memory (Kim et al., 2014), yet in the present study, slower printing did not appear to negatively impact the learning of new spellings. In contrast, laborious typing did have such a negative impact. This would suggest that there is something unique to typing that may affect cognitive and attentional resources more so than printing fluency does. At an elementary grade level, keyboarding can be more difficult than printing for some. Especially for children unfamiliar with keyboards, the other letters may serve as visual distracters and the child may expend more cognitive energy-and time- on visual scanning to find the right key. Anecdotally, our slower typers would often subvocalize while searching for the letter key (i.e., repeat the letter) which may have created even more interference within phonological working memory as well (for remembering the subsequent sounds). Due to the visual and phonological processes evoked in non-fluent typing, lack of typing proficiency may cause even more interference with attentional and cognitive resources than would weak printing, in turn detrimentally impacting spelling. It reasons then, that as children become more proficient with typing, they gain considerable speed, extraneous letters/sounds become less interfering and orthographic learning benefits. It is evident within the present results that the students varied considerably in their familiarity and comfort with the keyboard and this impacted learning; this concern can be traced back to when microcomputers were first introduced into classrooms (e.g., Varnhagen and Gerber, 1984). What is more surprising is that this concern is finding empirical support today.

The present study adds to the growing literature showing strong orthographic learning resulting from spelling practice (Conrad, 2008; Shahar-Yames and Share, 2008; Ouellette, 2010). The question remains, beyond individual modality differences, what explains the strong orthographic learning that occurs through spelling practice? Ouellette and Sénéchal (2008) have suggested that the benefit of spelling lies in its highly analytical nature that forces the child to consider each and every sound in a word. In producing the spelling, the child then must focus on each and every letter in their production. The result is that children attend to both the phonology and orthography of the word in more detail than they would need to during reading. Consequently, orthographic learning through spelling may result in representations that are more complete than would be created through reading (Conrad, 2008). As discussed by Perfetti and Hart (2002), while reading may proceed with partial representations, accurate spelling cannot. The analytic nature of spelling also promotes student engagement which can further benefit learning (Ouellette et al., 2013).

## Limitations, applications and directions for future research

The present study provides insight into the role of printing and typing in orthographic learning. A grade 2 sample was chosen as this represents a time where the transition to orthographic learning should be of particular relevance. However, it remains unclear whether these results are applicable at different grade levels. The methodology employed here lends itself well to future research with students at different grade levels, to trace the developmental progression of modality influences in spelling practice. In addition, while the modest size of the present sample is comparable to previous orthographic learning studies and sufficient for the number of steps in the regression models, replications with larger samples and with students of differing learning profiles will further advance knowledge in this area. Further, while the number of words per cell in our statistical analyses are modest, this is consistent with (actually greater than in) previous research that has employed an orthographic learning paradigm (e.g., Nation et al., 2007). Still, future research may wish to expand the non-word set and increase the orthographic complexity of stimuli used within this paradigm. Finally, it may be of interest in future research to explore word level evaluation of printing and typing skills. We found the complex sentence transcription task to be too difficult or abstract for this age group, but perhaps a task at the word level would add valuable insight into these developing skills; there may be as of yet unexplored lexical influence over printing not evident in printing isolated letters as is typically done in testing printing (see Kim et al., 2014). Likewise, future research may wish to qualitatively evaluate printing and hand-writing, to test for any possible role of quality over automaticity when it comes to literacy learning.

In summary, the current study assessed the effect of typing and printing on the orthographic learning garnered through spelling practice by grade 2 students. Results revealed no significant differences in learning between participants who practiced spelling the novel non-words by printing and those who practiced the non-words by typing. A hierarchical regression did reveal a significant role for pre-existing literacy skills, as well as an interaction between typing skill and practice modality. The present research is the first to employ an orthographic learning paradigm to compare the effects of typing vs. printing in literacy acquisition. The results do not support the hypothesis that the manual movements involved in printing make it a more effective learning modality, but instead highlight the importance of individual differences in learning and suggest that literacy draws upon modality-free lexical representations.

## Conflict of interest statement

The authors declare that the research was conducted in the absence of any commercial or financial relationships that could be construed as a potential conflict of interest.

## Footnotes

^1^The interaction reported for the recognition task reflected a similar pattern of results.

## Appendix

Target non-words and their alternatives.

**Table:**

**TARGET**	**HOMOPHONE**
**WORD LIST A**	
YAIT	YATE
LURT	LERT
VEAK	VEEK
WOTE	WOAT
ROOP	RUPE
**WORD LIST B**	
CALE	CAIL
VERN	VURN
ZEET	ZEAT
POAN	PONE
JUME	JOOM
